# Efficacy of tacrolimus monotherapy in primary membranous nephropathy

**DOI:** 10.1515/med-2024-0957

**Published:** 2024-05-28

**Authors:** Ya-pu Zhang, Lei Ran, Li Guo, Yan-Li Gou, Shan-shan Guo, Yang Xu, Xin Hua, Hang Chen

**Affiliations:** Department of Nephrology, Affiliated Hospital of Hebei University, Lianchi District, Baoding, 071000, Hebei, China; The Sixth Medical Center of Pla General Hospital, Haidian District, Beijing, China

**Keywords:** monotherapy, tacrolimus, primary membranous nephropathy, glucocorticoid, nephrotic syndrome, efficacy observation

## Abstract

**Objective:**

The aim of this study was to observe the remission of primary membranous nephropathy (PMN) and evaluate the efficacy of tacrolimus (TAC) monotherapy for PMN in comparison with TAC combined with a low-dose glucocorticoid (GC) protocol (TAC + GC).

**Methods:**

This was tested in a prospective monocentric observational trial of 70 patients with PMN, of whom 34 received TAC (0.05–0.075 mg/kg/day) or 36 received TAC (0.05–0.075 mg/kg/day) and GC (0.3–0.5 mg/kg/day of prednisone).

**Results:**

At 3, 6, 9, and 12 months of treatment, the effective rates in the TAC group and the TAC + GC group were similar (*P* > 0.05). The urinary protein quantification was reduced in patients under both therapeutic protocols, and the differences in the proteinuria quantification at 3, 6, 9, and 12 months of treatment were not statistically significant between the two groups (*P* > 0.05). The overall incidence of adverse reactions in the TAC group was lower than that in the TAC + GC group (23.5% < 36.1%), and the difference was statistically significant (*P* < 0.05).

**Conclusion:**

TAC monotherapy for PMN could effectively reduce urinary protein quantification and increase serum albumin levels. Compared with TAC + GC, TAC monotherapy for PMN had no difference in efficacy and fewer incidences of adverse reactions.

## 1 Introduction

Primary membranous nephropathy (PMN) is one of the most important causes of nephrotic syndrome in adults. The course of PMN is long and the treatment is difficult. As a result, PMN is an important cause of end-stage renal disease in adults. PMN has a high prevalence worldwide [1,2,3] and shows an annual increasing trend in China [4,5]. A previous study [6] has shown that PMN predominates in the middle-aged and elderly population, while a recent study [7] revealed that the disease tends to develop at a younger age in China. The therapeutic drugs for PMN include hormone combined with cyclophosphamide, cyclosporine, rituximab [8], tacrolimus (TAC), etc. [9].

TAC, a immunosuppressant that has been used to treat PMN in recent years, has differences in bioavailability among different populations. Differences in geography, ethnicity, and lifestyle habits can cause variations in efficacy, which may be correlated with genetic polymorphisms. Therefore, it is important to conduct studies on using TAC for PMN among patients from different geographical groups. In addition, the Kidney Disease: Improving Global Outcomes (KDIGO) guidelines state that TAC can be used either in combination with glucocorticoids (GCs) or as monotherapy [10]. The purpose of this study was to observe the efficacy of TAC in the treatment of PMN and to provide patients with a treatment plan with less side effects.

## 2 Materials and methods

### 2.1 Study subjects

After reviewing previous national and international related studies and the PMN treatment guidelines, the inclusion and exclusion criteria were formulated. From October 2018 to October 2019, 70 patients with PMN who were treated regularly at the Affiliated Hospital of Hebei University were selected as the study subjects. There were 23 cases of stage Ⅰ–Ⅱ MN, 31 cases of stage Ⅱ MN, and 6 cases of stage Ⅱ–Ⅲ MN, and renal biopsy was not performed in 10 cases. The reasons for the non-performance of renal biopsy in 10 patients were as follows: lack of patient consent for renal biopsy (9 cases) and presence of a solitary kidney (1 case).

#### 2.1.1 Inclusion criteria

Patients with a pathological diagnosis of PMN after a renal puncture biopsy or [10]; those diagnosed with PMN with positive serum anti-phospholipase A2 receptor (PLA2R) antibodies combined with clinical symptoms and strict exclusion of the secondary causes;

(1) Patients older than 18 years and younger than 70 years of any gender;
(2) Patients with a 24-h urine protein quantification of 4 g or more after 6 months of ARB/ACEI drug treatment, or those at intermediate risk of progressive renal injury according to a risk assessment [10] (the assessment criteria are detailed in Table 1);
(3) Patients with normal serum creatinine levels;
(4) Patients who did not receive other immunosuppressive therapy within the past 6 months;
(5) Patients with complete clinical data who could cooperate with regular follow-ups for 12 months;
(6) Patients who signed the informed consent form for the present study.

**Table 1:** Criteria for the evaluation of the risk for the progressive deterioration of renal function

Low risk	Intermediate risk	High risk	Very high risk
Normal eGFR; 24 h urine protein quantification below 3.5 g and/or serum albumin below30 g/l	Normal eGFR; 24 h urine protein quantification equals to or higher than 4 g or a decrease of less than 50% after proteinuria lowering therapy for 6 months; Mild proteinuria with low molecular weight; PLA2RAb lower than 50 RU/mL	Persist decrease in eGFR below 60 mL/min/1.73 m^2^ for more than 6 months, 24 h urine protein quantification >8 g; massive proteinuria with low molecular weight; PLA2RAb higher than 150 RU/mL	Life-threatening nephrotic syndrome; Rapid deterioration of renal function cannot be explained by other causes

Notes: eGFR: estimated glomerular filtration rate; PLA2RAb: M-type anti-phospholipase A2 receptor antibody. eGFR was calculated according to the CKD-EPI 2012 formula.

#### 2.1.2 The main exclusion criteria

(1) Patients with secondary membranous nephropathy;
(2) Patients with a decrease in kidney size as measured by ultrasound;
(3) Patients with abnormal hepatic function;
(4) Patients who were allergic to macrolides (TAC belongs to the macrolides);
(5) Patients with severe underlying disease, such as heart failure;
(6) Patients with serious complications, such as pulmonary embolism;
(7) Pregnant or lactating women and those preparing for pregnancy.

### 2.2 Methods

#### 2.2.1 Data collection and grouping

(1) General information was collected from all included subjects, including hospitalization ID, name, gender, age, and medical history.

(2) Baseline information was collected at the baseline time point with the initiation of treatment, and the following characteristics were included: symptoms and signs, systolic blood pressure (SBP), diastolic blood pressure (DBP), routine blood test, fasting blood glucose (FBG), total cholesterol (TCH), triglycerides (TG), glutamic aminotransferase (ALT), aspartate aminotransferase (AST), serum uric acid (UA), 24-hour urine protein quantification (24hUP), serum albumin (ALB), serum creatinine (Scr), estimated glomerular filtration rate (eGFR), and M-type PLA2R antibodies.

(3) The present study was a prospective study. Patients were grouped according to the random number table method, with 34 cases in the TAC group and 36 cases in the TAC combined with low-dose GC (TAC + GC) group.

### 2.3 Study methods

#### 2.3.1 General therapy

General symptomatic supportive treatments were given to patients in both groups, including dietary adjustments (to a low-salt diet), lipid regulation, urinary protein-lowering therapy, diuretics for those with edema, and anticoagulation therapy, after excluding any contraindications. Patients with low risk associated with anticoagulation and albumin levels of less than 3.0 g/dl were anticoagulated with low-molecular-weight heparin. Patients with an intermediate risk of anticoagulation-related bleeding and a serum albumin level of 2.0–2.9 g/dl are anticoagulated with low-molecular-weight heparin. For patients with LDL and high CVD risk, statins are used for lipid-lowering therapy.

#### 2.3.2 Specific treatment

TAC group: The initial dose of TAC was 0.05–0.075 mg/kg/day orally on an empty stomach, which was divided into two doses with an administration interval of 12 h. The blood trough TAC concentration was monitored regularly to adjust the drug dosage and maintained at 5–10 ng/mL.

TAC + GC group: The administration of TAC was the same as that in the TAC group. Additionally, 0.3–0.5 mg/kg/day of prednisone acetate tablets was administered once daily. After 2 months, the dose of prednisone acetate was gradually reduced with a reduction of 5 mg/day every 2 weeks until discontinuation of the drug.

#### 2.3.3 Dose reduction of TAC

TAC dose reduction was undertaken in patients in both groups after achieving sustained remission, and the dose was reduced to half of the initial dose from 4 to 8 weeks and maintained for 6–12 months.

### 2.4 Follow**-**ups

Regular follow-ups were conducted involving the collection and recording of the following information in patients in the two groups at 3, 6, 9, and 12 months of treatment: symptoms and signs, SBP, DBP, FBG, TCH, TG, ALT, AST, UA, 24hUP, serum ALB, and Scr.

### 2.5 Efficacy evaluation

The therapeutic efficacy was judged according to the standard of the 2012 KDIGO guidelines [11] (the criteria for the therapeutic efficacy are detailed in Table 2).

**Table 2:** The criteria for judging the efficacy for IMN after treatment

Complete remission (CR)	Partial remission (PR)	No remission (NR)
The disappearance of the clinical symptoms, negative for urine protein qualitative assay, 24 h urine protein quantification <0.3 g, serum albumin >35 g/L, normal serum creatinine level	The disappearance of the clinical symptoms, +∼++ in urine protein qualitative assay, 24 h urine protein quantification within 0.3–3.5 g; or a decrease of the 24 h urine protein quantification of more than half, serum albumin >30 g/L, stable renal function (an increase in the serum creatinine from the baseline level <20%)	Not meet the criteria for PR

The calculation of effective rate: The effective rate = (CR + PR)/the total number of cases × 100%. Whether with co-existence of hypertension. Note: By the Chi-square test, and the remaining was tested by the rank-sum test.

### 2.6 Adverse reactions

The occurrences of adverse reactions after drug administration were recorded. These mainly included gastrointestinal symptoms, elevated FBG, elevated blood pressure, infections, abnormal hepatic function, and abnormal renal function, among others.

### 2.7 Termination criteria of the study

(1) The occurrence of severe adverse reactions.
(2) The finding of a tumor during therapy.

### 2.8 Statistical analysis

**Figure 1:**
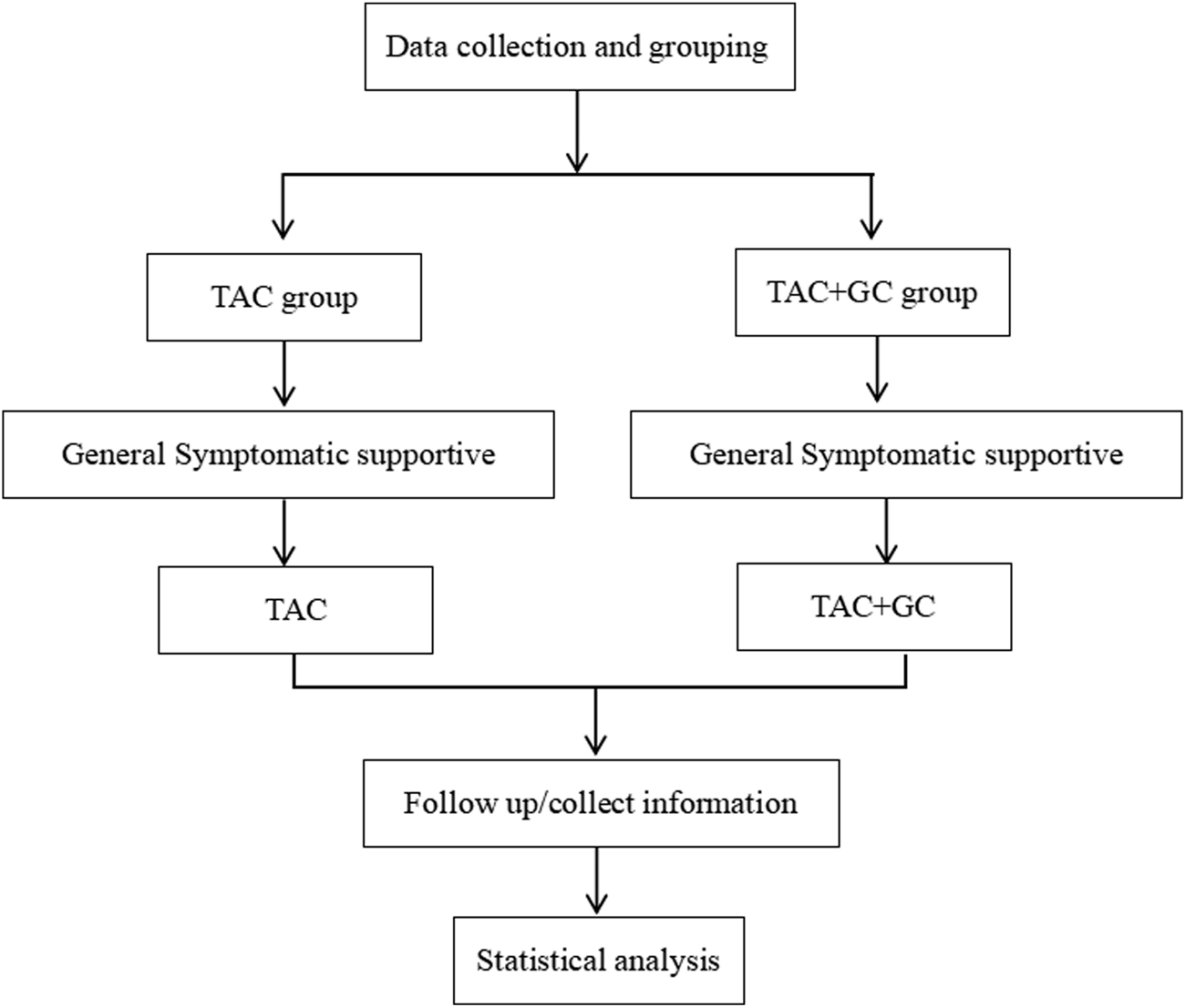
Flowchart of the study.


The SPSS Statistics 16.0 statistical software was adopted for data analysis and statistical processing for all data. The measurement data that satisfied the normal distribution were expressed as a mean ± standard deviation (*x* ± *s*), and the independent sample’s *t*-test was used for the comparison of differences between the groups. The measurement data that did not satisfy a normal distribution were expressed as the median and interquartile spacing M (P25, P75), and the rank-sum test was adopted for comparison of the differences between groups. The countable data were expressed as percentages, and the *X* ^2^ test was used to compare the differences between groups. *P* < 0.05 was considered to indicate statistical significance (Figure 1).

**Ethical approval:** This study was conducted in accordance with the declaration of Helsinki. This study was conducted with approval from the Ethics Committee of the Affiliated Hospital of Hebei University.
**Informed consent:** The patients/participants provided their written informed consent to participate in this study.

## 3 Results

### 3.1 Comparison of baseline characteristics between the two groups

The gender composition ratio (M/F) was 19/15 in the TAC group and 20/16 in the TAC + GC group. The age of the TAC group was 53 ± 17 years old and that of the TAC + GC group was 51 ± 19 years old. Both groups satisfied normal distributions. After statistical analysis, the data concerning the blood pressure, FBG, serum lipids, liver enzymes, Scr, serum ALB, 24hUP, and eGFR before the treatment did not satisfy the normal distribution in both groups. The differences in the general characteristics before the treatment were not statistically significant between the two groups (*P* > 0.05), and the data were comparable (as shown in Table 3).

**Table 3:** Comparison of baseline data between the two groups

Item	TAC group (*n* = 34)	TAC + GC group (*n* = 36)	*P*
Gender (male/female)	19/15	20/16	0.698
Age (years)	53 ± 17	51 ± 19	0.065
24UP (g/24 h)	6.37 (5.34, 7.27)	6.61 (5.35, 7.70)	0.552
ALB (g/L)	22 (20, 25)	21 (20, 22)	0.976
Scr (μmol/L)	63 (55, 74)	64 (56, 72)	0.144
TCH (mmol/L)	6.37 (5.44, 7.07)	6.51 (5.35, 7.14)	0.539
TG (mmol/L)	2.25 (1.76, 3.32)	2.36 (1.88, 3.17)	0.464
FBG (mmol/L)	5.0 (4.6, 5.3)	5.2 (4.7, 5.5)	0.763
SBP (mmHg)	111 (102, 119)	113 (105, 120)	0.114
DBP (mmHg)	74 (70, 78)	75 (71, 80)	0.657
eGFR (mL/min/1.73 m^2^)	98.1 (83.5, 111.8)	95.7 (81.4, 110.3)	0.248
PLA2RAb (+/−)	24/10	26/10	0.953
Hypertension or not (yes/no)	3/31	4/32	0.417

### 3.2 Comparison of the remission rate between the two groups

At 3 months of treatment, no cases reached complete remission in either group, and the effective rate in both groups was a partial remission rate. The effective rate was 17.6% in the TAC group and 19.4% in the TAC + GC group. The difference in the effective rate between the two groups was not statistically significant (*P* = 0.993, *P* > 0.05).

At 6 months of treatment, cases with complete remission appeared in both groups. In the TAC group, 2 cases (5.9%) achieved complete remission, 15 cases (44.1%) achieved partial remission. In the TAC + GC group, 2 cases (5.6%) achieved complete remission, and 17 cases (47.2%) achieved partial remission. The effective rate was 50.0% in the TAC group and 52.8% in the TAC + GC group. The difference in the effective rate between the two groups was not statistically significant (*P* = 0.967, *P* > 0.05).

At 9 months of treatment, in the TAC group, 4 cases (11.8%) achieved complete remission and 18 cases (52.9%) achieved partial remission. In the TAC + GC group, 5 cases (13.9%) achieved complete remission and 18 cases (50.0%) achieved partial remission. The effective rate was 64.7% in the TAC group and 63.9% in the TAC + GC group. The difference in the effective rate between the two groups was not statistically significant (*P* = 0.954, *P* > 0.05).

**Figure 2:**
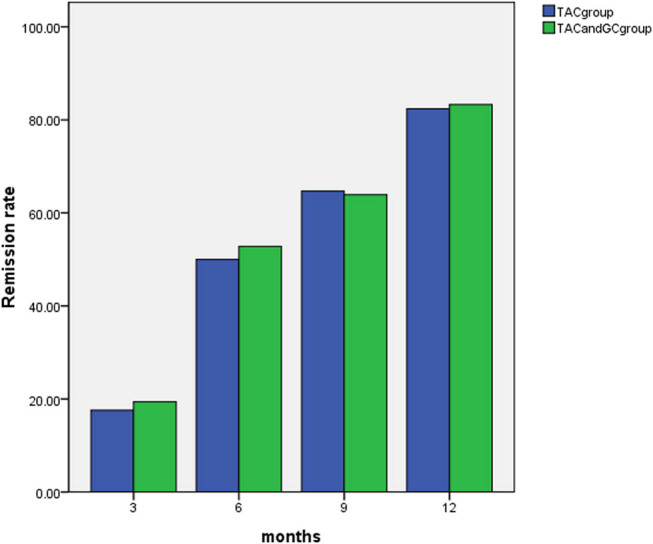
Comparison of the effective rates between the two groups.

At 12 months of treatment, in the TAC group, 14 cases (41.2%) achieved complete remission and 14 cases (41.2%) achieved partial remission. In the TAC + GC group, 14 cases (38.9%) achieved complete remission and 16 cases (44.4%) achieved partial remission. The effective rate was 82.4% in the TAC group and 83.3% in the TAC + GC group. The difference in the effective rate between the two groups was not statistically significant (*P* = 0.990, *P* > 0.05; as demonstrated in Table 4 and Figure 2).

**Table 4:** Comparison of efficiency between the two groups

Time	Curative effect	TAC group (*n* = 34)	TAC + GC group (*n* = 36)	*X* ^2^	*P*
3 months	CR	0	0		
	PR	6	7		
	NR	25	29		
	Efficiency	17.6	19.4	0.000	0.993
6 months	CR	2	2		
	PR	15	17		
	NR	17	17		
	Efficiency	50.0	52.8	0.068	0.967
9 months	CR	4	5		
	PR	18	18		
	NR	12	13		
	Efficiency	64.7	63.9	0.094	0.954
12 months	CR	14	14		
	PR	14	16		
	NR	4	5		
	Efficiency	82.4	83.3	0.114	0.990

PR: partial remission; CR: complete remission; NR: no remission.

### 3.3 Comparison of the 24hUP between the two groups

The 24hUP data in the two groups did not satisfy a normal distribution.

At 3 months of treatment, the 24hUP was 4.50 (3.46, 5.21) g in the TAC group and 4.34 (3.52, 6.06) g in the TAC + GC group. The difference in the 24hUP between the two groups was not statistically significant (*P* = 0.091, *P* > 0.05).

At 6 months of treatment, the 24hUP was 3.75 (2.94, 4.27) g in the TAC group and 3.59 (2.82, 4.35) g in the TAC + GC group. The difference in the 24hUP between the two groups was not statistically significant (*P* = 0.094, *P* > 0.05).

At 9 months of treatment, the 24hUP was 2.59 (1.34, 3.56) g in the TAC group and 2.63 (1.41, 3.72) g in the TAC + GC group. The difference in the 24hUP between the two groups was not statistically significant (*P* = 0.167, *P* > 0.05).

At 12 months of treatment, the 24hUP was 1.88 (0.25, 2.62) g in the TAC group and 1.62 (0.19, 2.54) g in the TAC + GC group. The difference in the 24hUP between the two groups was not statistically significant (*P* = 0.388, *P* > 0.05).

**Figure 3:**
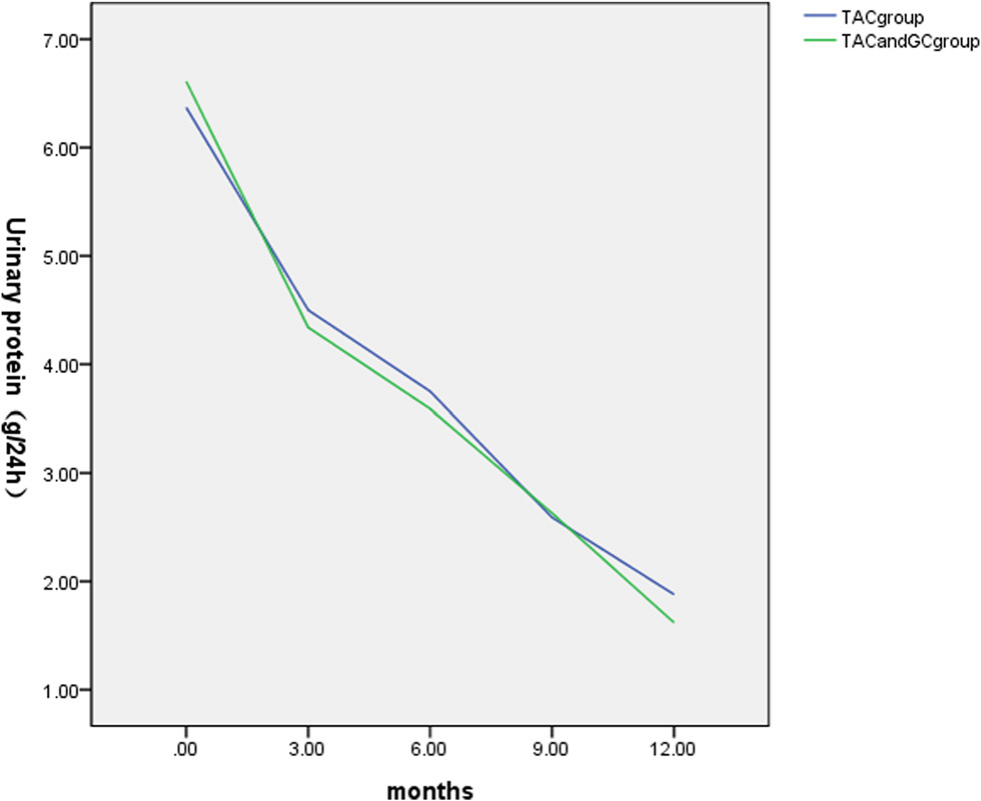
Comparison of the changes in the 24-h urine protein quantification between the two groups.

Compared with the observations before the treatment, the 24hUP decreased after the treatment at 3, 6, 9, and 12 months, and the differences were statistically significant within each group (*P* < 0.05; as illustrated in Table 5 and Figure 3).

**Table 5:** Comparison of urine protein quantification between the two groups

Time (months)	TAC group (*n* = 34)	TAC + GC group (*n* = 36)	*Z*	*P*
3	4.50 (3.46, 5.21)*	4.34 (3.52, 6.06)*	−1.693	0.091
6	3.75 (2.94, 4.27)*	3.59 (2.82, 4.35)*	−1.675	0.094
9	2.59 (1.34, 3.56)*	2.63 (1.41, 3.72)*	−1.381	0.167
12	1.88 (0.25, 2.62)*	1.62 (0.19, 2.54)*	−0.864	0.388

Note: The unit in 24 h urine protein quantification: g. *Compared with the same group before the treatment, *P* < 0.05.

### 3.4 Comparison of serum ALB levels between the two groups

The serum ALB data in both groups did not satisfy a normal distribution.

At 3 months of treatment, the serum ALB was 25 (23, 27) g/L in the TAC group and 23 (21, 24) g/L in the TAC + GC group. The difference between the two groups was not statistically significant (*P* = 0.097, *P* > 0.05).

At 6 months of treatment, the serum ALB was 33 (31, 37) g/L in the TAC group and 32 (29, 34) g/L in the TAC + GC group. The difference between the two groups was not statistically significant (*P* = 0.131, *P* > 0.05).

At 9 months of treatment, the serum ALB was 36 (33, 39) g/L in the TAC group and 35 (32, 39) g/L in the TAC + GC group. The difference between the two groups was not statistically significant (*P* = 0.986, *P* > 0.05).

At 12 months of treatment, the serum ALB was 36 (34, 41) g/L in the TAC group and 37 (35, 42) g/L in the TAC + GC group. The difference between the two groups was not statistically significant (*P* = 0.286, *P* > 0.05).

**Figure 4:**
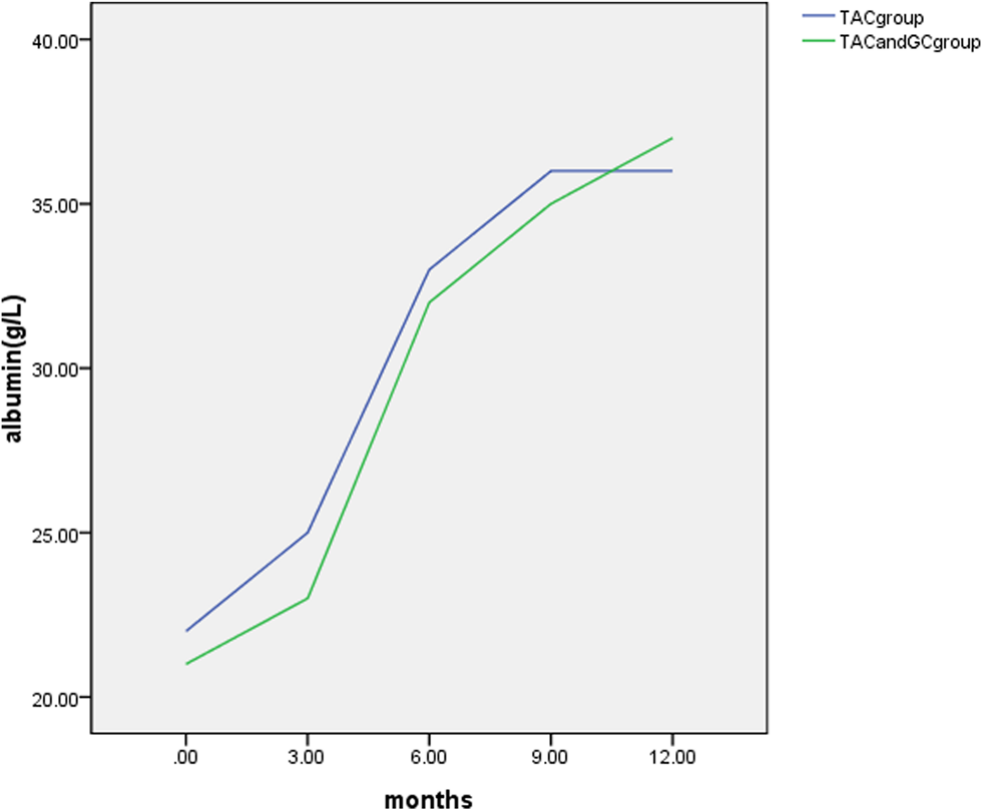
Comparison of the changes in serum albumin levels between the two groups.

Compared with the observations before the treatment, the serum ALB increased after the treatment at 3, 6, 9, and 12 months, and the differences were statistically significant within each group (*P* < 0.05; as demonstrated in Table 6 and Figure 4).

**Table 6:** Comparison of serum albumin levels between the two groups

Time (months)	TAC group (*n* = 34)	TAC + GC group (*n* = 36)	*Z*	*P*
3	25 (23, 27)*	23 (21, 24)*	−1.672	0.097
6	33 (31, 37)*	32 (29, 34)*	−1.509	0.131
9	36 (33, 39)*	35 (32, 39)*	−0.018	0.986
12	36 (34, 41)*	37 (35, 42)*	−1.068	0.286

Serum albumin unit: g/L. *Compared with before treatment, *P* < 0.05.

### 3.5 Comparison of Scr levels between the two groups

The Scr levels were within the normal reference range in both groups during the follow-ups, fluctuating between 60 and 70, and there was no significant increase or decrease at each follow-up time point.

### 3.6 Comparison of adverse reactions between the two groups

During the 12-month therapeutic period, there were 8 adverse drug reactions in the TAC group and 13 adverse drug reactions in the TAC + GC group.

In the TAC group, 4 gastrointestinal reactions occurred, and all subjects recovered after gastric acid suppression and stomach protection treatment. Infection occurred 3 times, and all subjects healed after anti-infection therapy. In the TAC + GC group, five gastrointestinal reactions occurred, and the symptoms improved after stomach protection treatments. Infection occurred four times, and all subjects healed after anti-infection therapy.

There was one case of hand tremor in each group.

In addition, two cases had increased FBG and one case had increased blood pressure in the TAC + GC group.

The total incidence of adverse reactions was lower in the TAC group than in the TAC + GC group (23.5% < 36.1%). The Chi-squared test showed that the difference was statistically significant (*P* = 0.031, *P* < 0.05; as shown in Table 7).

**Table 7:** Comparison of adverse reactions between the two groups

Item	TAC group (*n* = 34)	TAC + GC group (*n* = 36)	*X* ^2^	*P*
Increased fasting blood glucose	0	2		
Newly onset hypertension	0	1		
Gastrointestinal symptoms	4	5		
Infection	3	4		
Hand tremor	1	1		
An obvious increase in hepatic enzymes	0	0		
The incidence of adverse reactions	23.5%	36.1%	4.536	0.031

### 3.7 Comparison of recurrence between the two groups

The 18th month: one patient in the TAC group had recurrence after reaching PR, and the recurrence rate was 2.9% (1/34), which was adjusted to CTX + GC treatment; one patient in the TAC + GC group recurred in TAC reduction after reaching CR, and the recurrence rate was 2.8% (1/36), which was adjusted to TAC addition.

The 24th month: two patients in the TAC group recurred during TAC reduction after reaching PR, and the recurrence rate was 5.9% (2/34), which was adjusted to the CTX + GC regimen; in the TAC + GC group, three patients relapsed, and the recurrence rate was 8.3% (3/36). Two patients relapsed after reaching PR and were adjusted to CTX + GC regimen; one patient recurred during TAC reduction after CR, and TAC was added.

## 4 Discussion

In 2007, [12] the first randomized, controlled study of TAC monotherapy for PMN was conducted by Praga, a team of Spanish researchers. The study was a multicenter study that included 48 patients with PMN, who were divided into a TAC group (*n* = 25) and control group (*n* = 23), with symptomatic treatment being conducted in the control group. The results showed that the remission rate in the TAC group was over 80% after 12 months of treatment and over 90% after 18 months of treatment, while the remission rate in the control group receiving only supportive therapy was only 35%. It was the first time that the efficacy of TAC monotherapy for PMN was verified and supported by clinical data. However, an obvious limitation of that study was the small sample size and the fact that the control group in that study received only symptomatic supportive therapy without other types of immunosuppressive agents.

In 2010, Chen et al. [13], a multicenter, randomized, controlled trial of TAC for PMN, were conducted in China. Seventy-three patients with PMN were enrolled by Chen et al. The therapeutic effects were compared between the experimental group (the TAC group, *n* = 39) receiving TAC in combination with GC and the control group [the cyclophosphamide (CTX) group, *n* = 34] receiving CTX in combination with GC, with a follow-up of 12 months. The results showed that the remission rate in the TAC group was higher than that in the CTX group at 6 months of treatment. The remission rates in the two groups were similar at 12 months, indicating that the short-term efficacy of TAC was better than that of CTX, and the long-term efficacy was not inferior to that of CTX.

Subsequent studies have shown that TAC is effective in the treatment of PMN. Thus, the 2012 KDIGO guidelines make a clear recommendation for the application of TAC in the treatment of PMN. Since then, with the increase in related research, TAC has become an important focus in the study of PMN treatment, both nationally and internationally.

The results of the present study showed that the effective rates of TAC monotherapy at 3, 6, 9, and 12 months of treatment were 17.6, 50.0, 64.7, and 82.4%, respectively. Compared with the results of a randomized, controlled study by Praga, the remission rate at 12 months in Western patients with PMN treated by TAC alone was 82%. The remission rate at 12 months in the present study was similar to the above study, suggesting that TAC monotherapy has equally good efficacy in Chinese patients with PMN.

In comparison with a 2015 multicenter study by Caro et al. [14], the remission rate of TAC for PMN at 6 months was 60%, which was higher than the results in the present study, but the remission rate at 12 months in that study was 78%, which was slightly lower than that in the present study. It should be noted that a sample of 122 cases was included in the study conducted by Caro et al., which was a larger sample size than that in the present study, and might therefore differ slightly in the remission rate, but the difference was not significant.

The results of the present study were compared with a related study with a larger sample size conducted by Qin et al. [15] in China. The results of the mentioned study revealed that the remission rate in 408 patients with PMN at 6 months was 50%, which was consistent with the results of the present study, and the remission rate at 12 months was 63%, which was significantly lower than that at 12 months in the present study. In addition, the results of the present study showed that the effective rates of TAC combined with low-dose GC treatment at 3, 6, 9, and 12 months were 19.4, 52.8, 63.9, and 83.3%, respectively, and the complete remission rates were 0.0, 5.6, 13.9, and 38.9%, respectively. The results were similar when compared with a randomized, controlled study [16] in which TAC was used in combination with low-dose GC for PMN and achieved an overall remission rate of 71.0% at 12 months.

The results were more similar when compared with a study conducted by researchers from Shandong University in 2019 [7], in which TAC was used in combination with low-dose GC and achieved an overall remission rate of 56.5% at 6 months and 80.4% at 12 months. In addition, the complete remission rates at 6, 9, and 12 months in the TAC monotherapy group in the study from Shandong University were significantly higher than the complete remission rates in the present study. After a further comparative analysis of the screening conditions of the study subjects, it was found that the subjects selected in the above study were young patients with PMN, aged between 15 and 40 years, who might have had a better average physical fitness than those included in the present study and therefore achieved a higher complete remission rate in a shorter period (6 months). Combined with other recently published studies [17,18,19], the remission rate of TAC monotherapy for PMN between 6 and 12 months was approximately 60–80% and that of TAC combined with low-dose GC for PMN between 6 and 12 months was 55–80%. Therefore, there was no significant difference in the remission rate between TAC monotherapy and TAC combined with low-dose GC protocol.

In addition, the TAC monotherapy protocol has been shown to have some advantages over or to have comparable efficacy to the protocols of TAC combined with other drugs, except for low-dose GC, in the treatment of PMN. In 2019, a study [20] compared the difference in efficacy between TAC monotherapy and CTX combined with GC at different time points in the treatment of PMN. It was shown that TAC monotherapy achieved a higher complete remission rate than that in the CTX group at 3 months of treatment, and after 6 months, TAC and CTX combined with GC achieved comparable remission rates. A relevant meta-analysis [21,22,23,24] revealed a higher remission rate in the TAC-treated group than in the CTX-treated group. Therefore, the short-term efficacy of TAC may be better than that of CTX, but the long-term efficacy of TAC might not be outstanding. This could be because CTX should be applied at a certain cumulative dose to achieve significant efficacy. Thus, TAC has an advantage in the short-term treatment.

In 2017, the remission rates of TAC monotherapy versus CTX in combination with GC for PMN were compared and analyzed in a randomized, controlled study by Liang et al. [25]. The results showed no significant difference in the remission rate between the two protocols at 12 months of treatment. Therefore, we believe that comparable efficacy could be achieved by applying TAC to patients with PMN who were unwilling to receive alkylates.

Regarding adverse reactions, during the 12-month treatment and follow-up period in the present study, there were 8 adverse drug reactions (23.5%) in the TAC group and 13 adverse drug reactions (36.1%) in the TAC + GC group. Among the adverse reactions, the incidence of infection was the highest, probably because of the decrease of albumin in patients with PMN and the decrease of the immune function resulting from the administration of immunosuppressive drugs. In a recent study [26], the efficacy and adverse effects of GC monotherapy were compared with those in patients treated with GC plus TAC for PMN. It was found that the incidence of adverse effects in GC combined with TAC was not higher than that in the GC monotherapy. Some related meta-analyses [27,28] confirmed that TAC resulted in fewer adverse effects, such as infections, abnormal glucose tolerance, liver function impairment, or decreased white blood cells than those with CTX.

TAC alone is effective in the treatment of PMN, but it still has a recurrence rate. 47% of patients relapsed 18 months after discontinuation of TAC [10]. Relapse rate after any remission was 40% in the TAC + GCs group [29]. In our study, at the 24th month, the recurrence rate was 5.9% (2/34) in the TAC group and 8.3% (3/36) in the TAC + GC group. TAC alone and increasing the blood concentration of TAC still had a certain effect in relapsed CR patients. Patients in the TAC long-course group (24 months of treatment) had higher remission rate and lower recurrence rate [30]. This suggests that a slower reduction and withdrawal of TAC may reduce the recurrence rate. However, whether the long-term benefit of TAC monotherapy for PMN patients is more obvious needs to be further confirmed by long-term follow-up randomized controlled studies.

## 5 Conclusion

In summary, in the treatment of PMN, TAC monotherapy achieved an effective rate that was indistinguishable from that in the TAC combined with a low-dose GC protocol, both in the short term (3 months) and the long term (12 months). Both protocols were effective in reducing urinary protein quantification and elevating serum ALB. Moreover, TAC monotherapy had a lower incidence of adverse reactions and was safer than the combined GC protocol. However, this test has some limitations.

This test is a single-center clinical observation, with limited sample size and certain selection bias. We recruited only patients younger than 70 years and only those with normal serum creatinine levels.

In this test, the serum anti-PLA2R antibody titer was detected only at the initial visit, but the change of this index was not detected in the later follow-up, and the sequence and correlation between the decrease in anti-PLA2R antibody titer and the remission of clinical indexes were not further discussed.

We did not monitor the lymphocyte subsets and did not discuss the correlation between CD19 + depletion/replication and treatment response. We attached great importance to it in the later stage.

PMN is a drawn-out disease. In the TAC group, one case relapsed in the 18th month and two cases recurred in the 24th month. In the TAC + GC group, one case relapsed at the 18th month and three cases relapsed at the 24th month. At the 24th month, the recurrence rate was 5.9% (2/34) in the TAC group and 8.3% (3/36) in the TAC + GC group; thus, further clinical observation should be necessary for the evaluation of the long-term efficacy and prognosis.
